# The alkylphospholipid, perifosine, radiosensitizes prostate cancer cells both *in vitro *and *in vivo*

**DOI:** 10.1186/1748-717X-6-39

**Published:** 2011-04-15

**Authors:** Yuanhong Gao, Hiromichi Ishiyama, Mianen Sun, Kathryn L Brinkman, Xiaozhen Wang, Julie Zhu, Weiyuan Mai, Ying Huang, Daniel Floryk, Michael Ittmann, Timothy C Thompson, E Brian Butler, Bo Xu, Bin S Teh

**Affiliations:** 1 Department of Radiation Oncology, The Methodist Hospital Research Institute, Weill Cornell Medical College, Houston, TX 77030, USA; 2 Department of Radiology/Radiation Oncology, Baylor College of Medicine, Houston, TX 77030, USA; 3 Michael E. DeBakey VA Medical Center, Houston, TX 77030, USA; 4 The State Key Laboratory of Oncology in Southern China, Guangzhou, China; 5 Sun Yat-Sen University Cancer Center, Guangzhou, China; 6 Department of Radiology and Radiation Oncology, Kitasato University School of Medicine, Sagamihara, Kanagawa, Japan; 7 Cancer Hospital, Chinese Academy of Medical Sciences and Peking Union Medical College, Beijing, China

## Abstract

**Background:**

Perifosine is a membrane-targeted alkylphospholipid developed to inhibit the PI3K/Akt pathway and has been suggested as a favorable candidate for combined use with radiotherapy. In this study, we investigated the effect of the combined treatment of perifosine and radiation (CTPR) on prostate cancer cells *in vitro *and on prostate cancer xenografts *in vivo*.

**Methods:**

Human prostate cancer cell line, CWR22RV1, was treated with perifosine, radiation, or CTPR. Clonogenic survival assays, sulforhodamine B cytotoxity assays and cell density assays were used to assess the effectiveness of each therapy *in vitro*. Measurements of apoptosis, cell cycle analysis by flow cytometry and Western blots were used to evaluate mechanisms of action *in vitro*. Tumor growth delay assays were used to evaluate radiation induced tumor responses *in vivo*.

**Results:**

*In vitro*, CTPR had greater inhibitory effects on prostate cancer cell viability and clonogenic survival than either perifosine or radiation treatment alone. A marked increase in prostate cancer cell apoptosis was noted in CTPR. Phosphorylation of AKT-T308 AKT and S473 were decreased when using perifosine treatment or CTPR. Cleaved caspase 3 was significantly increased in the CTPR group. *In vivo*, CTPR had greater inhibitory effects on the growth of xenografts when compared with perifosine or radiation treatment alone groups.

**Conclusions:**

Perifosine enhances prostate cancer radiosensitivity *in vitro *and *in vivo*. These data provide strong support for further development of this combination therapy in clinical studies.

## Background

Prostate cancer currently remains the most commonly diagnosed malignancy and is second only to lung cancer as the leading cause of tumor related death in males [1]. Radiotherapy (including external beam radiotherapy and brachytherapy) remains a very important treatment modality for prostate cancer. However, prostate cancer cells can easily become radioresistant, resulting in poor long term prognosis for many prostate cancer patients. Therefore, it is now essential to clarify and target underlying mechanisms involved in the development of radioresistant cells to improve and optimize radiotherapy strategies for prostate cancer patients.

Many molecular targets are differently expressed between tumor and normal tissue types. This offers the possibility of specific, biology-driven modulation radiation responses in tumor and normal tissue types, and thereby a therapeutic gain. In particular, the epidermal growth factor receptor (EGFR) family has been targeted to overcome radiation resistant cancer cell types [2]. The EGFR-activated phosphatidylinositide 3-kinase/Akt (PI3K/Akt) pathway has been proposed to protect cells from radiation-induced apoptosis by multiple mechanisms [3]. Deregulation of the PI3K/Akt pathway is often associated with tumorigenesis [4,5] and poor prognosis in cancer patients [6-8]. In addition, the PI3K/Akt pathway has been implicated extensively as a contributor to radioresistance [9]. These insights present the PI3K/Akt pathway as an attractive target for anticancer therapy, and more importantly, for combined treatment therapy.

Perifosine is an orally applicable, membrane-targeted alkylphosphocholine analogue with antitumorigenic activity and has been found to effectively inhibit Akt in preclinical models. Other alkylphospholipids have already been found to exhibit radiosensitizing properties when used to treat squamous cell carcinoma [10-12] malignant glioma [13], and lymphoma [14]. However, the effect of alkylphospholipids on prostate cancer cells has yet to be fully investigated. The results of a recent Phase I/II clinical trial of perifosine failed to show significant therapeutic response when used as a single agent [15]. However, Vink et al. [16] suggest that alkylphospholipids, including perifosine, are attractive candidates for combination treatment with radiotherapy.

The aim of this study was to investigate the effect of the combined treatment of perifosine and radiotherapy on human prostate cancer.

## Methods

### Cell culture

The human prostate adenocarcinoma cell line, CRW22RV1 [17] was cultured in RPMI 1640 containing 25 mM HEPES buffer, L-glutamine, 50 units/ml penicillin, 50 μg/ml streptomycin and 10% fetal bovine serum in a humidified incubator set to 37°C, 5% CO_2_. The cells were plated and cultured to achieve 80-90% confluence on the day of experiments.

### Radiation

For *in vitro *experiments, cells were irradiated at a dose rate of 2.10 Gy per minute using the GAMMATOR B Cs-137 irradiator (Radiation Machinery, Parsippany, NJ). For *in vivo *experiments, mice were immobilized with durative anesthesia by inhalation using the Table Top Anesthesia Machine (VetEquip, Inc., Pleasanton, CA) and a custom designed flake of plumbum, which allows for specific radiation of a subcutaneous tumor while shielding the rest of the animal. Xenografts were irradiated at a dose rate of ~1.56 Gy per minute using a Phillips X-ray machine.

### Perifosine treatment

Perifosine was purchased from Selleck Chemicals LLC. For cell proliferation assays, cells were incubated from 24 to 144 hours with 10 μM perifosine. For measurements of apoptosis, cells were incubated for 24 hours with 10 μM perifosine. For clonogenic survival assays, cells were incubated for 48 hours with 15 μM or 30 μM perifosine.

### Cell proliferation assays

Cell viability was determined with a colorimetric 3-(4,5-dimethylthiazol-2-yl)-5-(3-carboxymethoxyphenyl)-2-(4-sulfophenyl)-2H-tetrazolium assay (MTS; Promega, Madison, WI). Cells were seeded at a density of 5000 cells per well in 96-well plates. Immediately after perifosine treatment, cells were treated with 6 Gy of radiation. After treatment with perifosine for 24, 48, 72, 96, 120, or 144 hours, 20 μL of MTS reagent was added to each well. Two hours later, optical absorbance was measured at 490 nm. Experiments were performed in triplicate and repeated at least 3 times.

### Clonogenic survival assays

Cells (200-10,000) were plated in 6-cm diameter dishes and incubated 4 hours to allow the cells to attach. Cells were then treated with perifosine and immediately thereafter with 2 - 8 Gy of radiation. After 48 hours, perifosine was removed and replaced with fresh medium. Cells were allowed to form colonies over a period of 14 days after treatment, which were subsequently fixed and stained by 0.2% crystal violet. The number of colonies containing at least 50 cells was determined under a light microscope. The plating efficiency was calculated by the number of colonies/cells seeded. The surviving fraction at each dose was determined as a ratio of plating efficiencies for irradiated and non-irradiated cells, in which 100% corresponded to the non-irradiated control for each group. The survival curves were plotted by linear regression analyses. A D_0 _value, representing the radiation dose that leads to 37% of cell survival, was calculated. Sensitizing enhancement ratios (SER) were then calculated based on the D_0 _values according to the following formula.

### Apoptosis measurement

Cells (1.2 × 10^5^) were seeded in 6-cm diameter dishes and incubated overnight to allow the cells to attach. Cells were then treated with perifosine and immediately thereafter with 6 Gy of radiation. Twenty-four hours later, the media was replaced with fresh media. To avoid losing apoptotic cells, supernatants were centrifuged and cells in the media were collected and stored for further study. An additional 24 hours later, cells and supernatants were collected, washed, and resuspended in Nicoletti buffer. Apoptotic cells were measured by fluorescence activated cell sorting (FACS) after Annexin-FITC and propidium iodide (PI) double staining using the Annexin V Apoptosis Detection Kit, according to the manufacturer's protocol (BD, Franklin Lakes, NJ). The percentages of apoptotic cells were analyzed using FACScaliber software programs. Experiments were repeated 3 times.

### SDS-page and western blot analysis

Primary monoclonal antibodies against total AKT, phosphorylated AKT (Ser473 and Thr308) and cleaved caspase 3 (Asp175) were purchased from Cell Signaling Technologies (Beverly, MA). Antibodies against β-actin were obtained from Chemicon (Temecula, CA). Horseradish peroxidase-conjugated secondary antibodies were obtained from Santa Cruz Biotechnology (Santa Cruz, CA). Total protein was extracted from cells using cell lysis buffer (Cell Signaling Technology). Cells were harvested in 4°C lysis buffer (150 mM NaCl, 20 mM pH 7.5 Tris-HCl, 1% NP40, 1 mM EDTA) supplemented with protease cocktail (Roche, Indianapolis, IN) and phosphatase I and II inhibitors (Sigma, St. Louis, MO) on ice. Following centrifugation at 14,000 rpm for 10 minutes at 4°C to remove the insoluble fraction, protein concentrations of the supernatants were determined by BCA assay (Pierce, Rockford, IL). Cell lysates were mixed with Laemmli sample buffer and placed in a boiling water bath for 5 min. Equal amounts of protein (20 μg/lane) were loaded into 10% sodium dodecyl sulfate-polyacrylamide gels (Invitrogen, Carlsbad, CA) and separated by electrophoresis. Protein was then transferred electrophoretically onto nitrocellulose membranes (Bio-Rad, Hercules, CA). The membranes were blocked in 5% skim milk in TBS-T (500 mM NaCl, 20 mM pH 7.5 Tris-HCl, 0.1% Tween 20) and incubated overnight at 4°C. The membranes were probed with primary antibodies and secondary antibodies according to the manufacturer's instructions. The blots were analyzed by chemiluminescence detection and autoradiography.

### In vivo tumor growth delay assays

All animal studies were conducted in compliance with VA Medical Center Animal Care and Use policy. Male Athymic Nude-Foxn1nu mice, 6 to 7 weeks old (19.8-26.5g), were purchased from Harlan Laboratories, Inc. (Indianapolis, Indiana). Animals were kept and handled under a 12h/12h light/dark cycle at 22°C, received a standard diet and acidified water. Mice were given subcutaneous injections of 5 × 10^6 ^cells in 100 μl HBSS into the right hind limb and tumor size was measured using calipers at least two times per week. Tumor volume was calculated as π/6 × length × width × height, where tumor volume at the start of treatment was normalized to 100%. When tumors had grown to an average volume of 100 mm^3^, mice were separated into 4 groups: control (no perifosine, shame-irradiated, n = 10), perifosine (oral perifosine, n = 10), radiotherapy (local tumor radiation, n = 9), and combined therapy (oral administration of perifosine and local tumor radiation, n = 11). Perifosine and combined groups were given perifosine in a loading dose of 300 mg/kg (2 × 150 mg/kg separated by 12 hours) followed by daily maintenance doses of 35 mg/kg for 5 days. Two fractions of 5 Gy radiation were delivered the next day and 4 days after the start of perifosine treatment.

## Results

### Perifosine increases sensitivity of human CWR22RV1 cells to radiation

**Figure 1:**
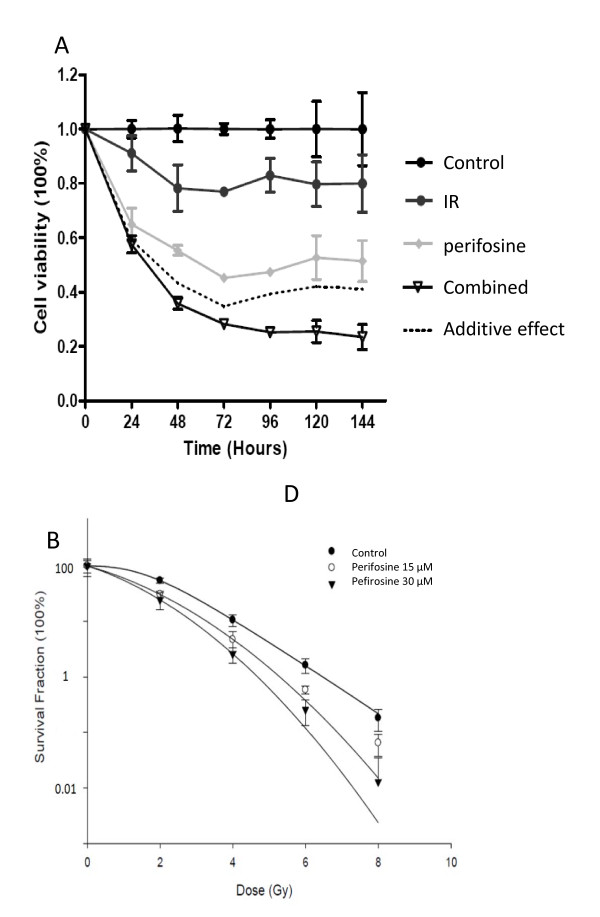
**Perifosine increases prostate cancer radiosensitivity in vitro**. ***A***, CWR22RV1 cells were irradiated in the absence (control) or the presence of 10 μM perifosine for 24 hours and the cell viability was assessed using MTS assay. Shown are the means and standard deviation of each individual treatment points. ***B***, Cells were irradiated in the absence (control) or in the presence of 15 μM and 30 μM perifosine and the colony formation assay was conducted. Shown are the means and standard deviation of each individual treatment points.

In order to assess the effect of perifosine on prostate cancer radiosensitivity, we first tested various doses of perifosine exposure in combination with radiation treatment in CWR22RV1 cells using the proliferation assay (MTS assay) and the colony formation assay. We found that the combination of perifosine and radiation had a greater inhibitory effect on cell viability compared to perifosine or radiation alone (Figure 1A). Similarly, the combination of perifosine and radiation had a greater inhibitory effect on colony formation compared to perifosine or radiation alone (Figure 1B). The sensitization enhancement ratios (SER) calculated based on the D_0 _value from 15 μM and 30 μM perifosine were 1.47 and 1.78, respectively. It is noted that for the survival curves plotted, combinational survival was normalized by the effect of perifosine alone on survival. The result of the colony formation assay was confirmed in the prostate cancer cell line PC-3 (Additional File 1, Figure S1).

### Perifosine on radiation induced apoptosis and cell cycle arrest

**Figure 2:**
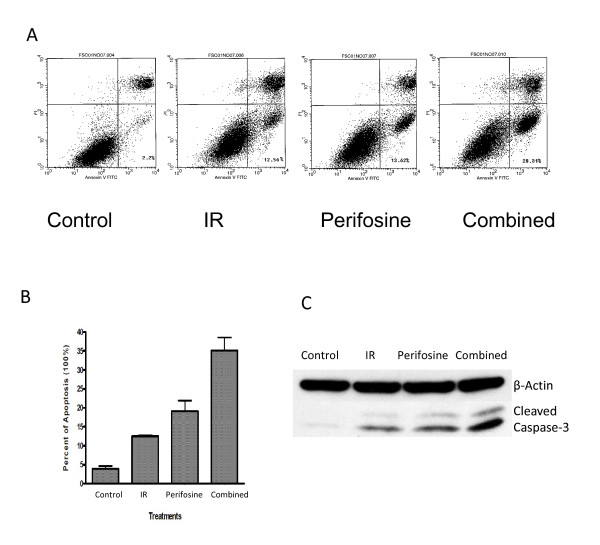
**Effects of perifosine on radiation-induced apoptosis and the G2/M checkpoint**. ***A***, CWR22RV1 cells were treated with perifosine (10 μM), radiation (6Gy, IR), or combination as indicated. Cellular apoptosis was detected by FACs. **B**. Quantititative analysis of the FACs data. **C**. CWR22RV1 cells were treated with control, radiation only (6Gy, IR), perifosine only (5 μM) or combination before they were subjected to the Western blot analysis using indicated antibodies.

To assess the effect of perifosine on radiation-induced apoptosis, we used Annexin-FITC based flow cytometry analysis. Both nuclear fragmentation with propidium iodine (PI) staining and translocated membrane phosphatidylserine (PS) with Annexin V staining were measured. Cells in early apoptosis shown in the right lower quadrant were regarded as apoptotic cells (Figure 2A). We found that both perifosine and radiation induced significant apoptotic responses as shown by the increase of apoptotic cell (Figure 2B). When radiation (6Gy) and perifosine (10 μM) were combined, the number of apoptotic cells was significantly increased (Figure 2B). This apoptosis result was also confirmed in the prostate cancer cell line PC-3 (Additional File 1, Figure S2). We also found that the level of cleaved caspase 3 was the highest in the combined treatment group (Figure 2C), indicating a potential mechanism of radiosensitization. We also analyzed cell cycle checkpoints induced by perifosine, radiation, or the combination using propidium iodine (PI) staining followed by flow cytometry analysis. We found that perifosine alone did not induce cell cycle arrest at the G2/M phases and perifosine did not affect the IR-induced G2/M checkpoint (data not shown). These observations indicate that perifosine induced radiosensitization is independent of the G2/M checkpoint.

### Effects of perifosine on PI3K/Akt activity

**Figure 3:**
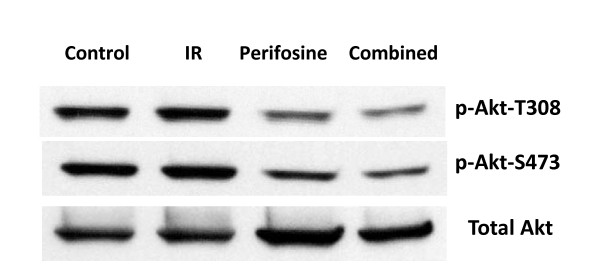
**Perifosine and Akt activity**. CWR22RV1 cells were treated with control, radiation only (6Gy, IR), perifosine only (5 μM) or combination before they were subjected to the Western blot analysis using indicated antibodies.

To determine the effect of the combination of perifosine and radiation on Akt activity, we assessed expression levels of phospho-Akt-Thr308 and phospho-Akt-Ser473 by Western blot. We found that while the radiation-only group did not affect Akt-T308p and S473p, perifosine significantly reduced phosphorylation of Akt (Figure 3). More interestingly, combination of radiation with perifosine further reduced Akt phosphorylation, suggesting a synergistic inhibitory effect of perifosine and radiation on AKT phosphorylation. Since phosphorylation of Akt is linked to Akt activity, our results indicate that combination of perifosine with radiation can significantly increase the inhibitory effect of perifosine on Akt.

### Perifosine enhances prostate cancer radiosensitivity in vivo

**Figure 4:**
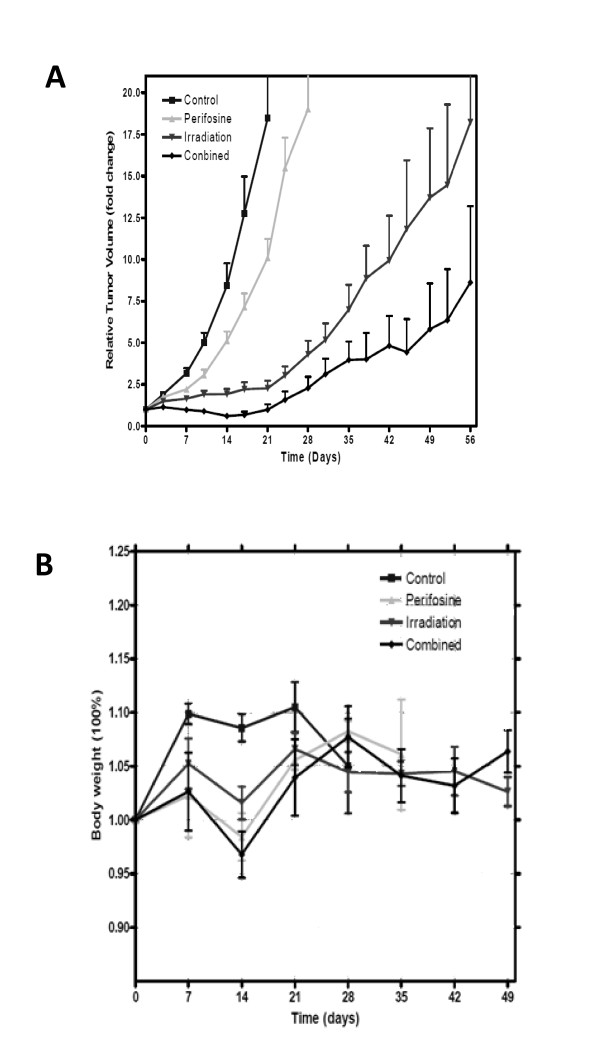
**Perifosine radiosensitizes prostate cancer *in vivo***. **A**. Nude mice bearing CWR22RV1 xenografts with a mean volume of 100 mm^3 ^were treated with control, perifosine alone, radiation alone or combination. The tumor size was measured at least two times a week and the tumor growth delay curve was displayed. **B**, Changes of body weight after treatment.

We then investigated the *in vivo *radiosensitization effect of perifosine in a prostate cancer xenograft model in nude mice. Perifosine treatment protocols in the clinical setting typically involve an initial loading dose followed by daily maintenance doses. Therefore, in an attempt to simulate the clinically relevant treatment protocol, we delivered perifosine as a loading dose followed by five daily maintenance doses. Specifically, animals bearing prostate cancer were given perifosine in an initial dose of 300 mg/kg (2 × 150 mg/kg separated by 12 hours) followed by daily maintenance doses of 35 mg/kg for 5 days. This perifosine treatment protocol was shown to result in similar perifosine levels and pharmacokinetics as in humans[16]. We found that perifosine alone did not have a significant effect on tumor growth. However, perifosine can significantly increase radiation induced tumor growth delay (Figure 4A and Additional File 1Figure S3). To reach the 10-fold size of tumor volume to the initial volume in the control, it took 15, 19, 41 and 59 days in control, perifosine only, radiation only and combined treatment groups, respectively. It is noted that in one case, the combined treatment led to a complete remission of the CWR22RV1 tumor.

We also measured toxicity after irradiation and oral perifosine treatment. The body weight of the nude mice was monitored and used as an index for assessing the systemic toxicity. In all experimental groups, no significant weight loss due to local tumor irradiation was observed. Body weight of control mice increased ~10% within the first week, and then maintained this level for two weeks. After the fourth week, mice lost ~5% body weight due to dyscrasia. Perifosine alone resulted in a slight but reversible weight loss (~5%), which was sustained for 10 days. A reduction in body weight of ~6% was observed in the combination group during the second and third weeks. However, this weight loss was reversible, as the body weight was regained within 3 weeks (Figure 4B). No lethal dose effect was observed.

## Discussion

In this study, we showed enhancement of radiation-induced cell death by the alkylphospholipid perifosine in CWR22RV1 prostate cancer both *in vitro *and *in vivo*. *In vitro*, perifosine reduced cell viability and clonogenic survival, and enhanced apoptosis after radiation. *In vivo*, substantial tumor growth delay was observed when perifosine was combined with radiation.

As a single agent, perifosine has been reported to have limited antitumor activity [18,19]. However, the combination of classical anticancer regimens with novel biological response modifiers has potential to modulate signal transduction pathways mediating apoptosis, proliferation, and survival. Perifosine is therefore a rational candidate for combined modality approaches [2,11,20]. Indeed, perifosine has demonstrated (supra-) additive cytotoxicity *in vitro *when combined with other drugs [21-24]. In addition, several alkylphospholipids have been shown to enhance radiation-induced cell death in a variety of tumor types *in vitro *[10,11,14,20,25]. The following are possible mechanisms of Akt inhibition by perifosine that have been suggested: 1) perifosine disrupts the structure of and signaling within lipid rafts, preventing Akt recruitment to the membrane, 2) perifosine binds directly to and inhibits the pleckstrin homology (PH) domain of Akt [19]. In our study, reduced phospho-Akt-T308 and phospho-Akt-S473 were observed in perifosine alone and the combination groups, indicating radiation combed with perifosine can increase the inhibitory effect of perifosine on Akt, resulting in a synergistic effect.

Although Akt plays an important role in the mechanism by which perifosine exerts its antitumor effect, Akt is clearly not the only molecule involved. Other potential targets may include stimulation of the cellular stress-related, apoptosis-inducing SAP/JNK pathway [14,26]; stimulation of FAS clustering [27]; inhibition of the MAP/ERK pathway [28]; inhibition of phospholipase C [29] and protein kinase C activation [30]; and stimulation of ceramide formation [31]; and phospholipase D [31,32]. At this time, further studies are needed to confirm other pathways involved in the antitumor effect of combined perifosine and radiation treatment of prostate cancer cells.

Hilgard et al. reported that a single oral (loading) dose therapy with high-dose perifosine (68.1 mg/kg) caused inhibition of tumor growth for about 14 days, and daily oral treatments (for 25 days) at lower doses (2.5 to 46.4 mg/kg) also caused tumor growth inhibition. The onset of response was found to be dose related. Responses persisted for > 20 days after termination of therapy without clear dose-response relationships over this range [33]. Based on these results, a loading dose followed by a lower daily maintenance dose schedule was used in this study. Many Phase I/II studies have also used a loading dose followed by maintenance dose schedules, with reported loading doses ranging from 300 mg/kg to 1050mg/kg and maintenance doses ranging from 50 mg/kg to 150 mg/kg [16]. Thus, we decided to use 300 mg/kg for loading doses and 35mg/kg for daily maintenance doses.

Vink et al. demonstrated complete and sustained tumor regression of xenografted squamous cell carcinoma after combined treatment of radiation and perifosine [12]. Their schedule was based on daily doses without loading doses. Although they demonstrated complete tumor regression using a combination of 3 × 40 mg/kg perifosine and 2 fractions of 5 Gy radiation daily, our study could not achieve complete regression, even when combining a 300 mg/kg perifosine loading dose with 5 × 35 mg/kg perifosine and 2 fractions of 5 Gy radiation daily. Variation between our results and previous results are likely caused by the differences in radiosensitivity between squamous cell carcinoma and prostate cancer cells, in addition to the differences between schedules of drug administration. Further studies should be performed to determine the best treatment schedule for future clinical studies.

## Conclusions

In conclusion, perifosine enhances prostate cancer radiosensitivity, as evidenced by reduction of cell viability, clonogenic survival, and the increase of apoptosis *in vitro *and by tumor growth delay *in vivo*. These data provide strong support for further development of this combination therapy in clinical studies.

## Competing interests

The authors declare that they have no competing interests.

## Authors' contributions

YG and BT designed the study, collected the data, interpreted the results of the study, performed the statistical analysis and drafted the manuscript. BX and BT oversaw the project completion, analyzed the data and completed the manuscript. HI, MS, KB, XW, JZ, WM, YH, DF, MI participated in experimentation and data acquisition. TT and EB contributed to reagents and participated in discussions. All authors read and approved the manuscript.

## Supplementary Material

Additional file 1 **Figure S1: Radiosensitization of perifosine in prostate cancer PC-3 cells**. Cells were irradiated in the absence (control) or in the presence of perifosine and the colony formation assay was conducted. Shown are the means and standard deviation of each individual treatment points. Figure S2: Perifosine and radiation induced apoptosis in PC-3 cells. Cells were treated with perifosine (5 μM), radiation, or combination as indicated. Cellular apoptosis was detected by FACs. Shown are the mean values of the quantitative data. Figure S3: Perifosine increases radiation induced tumor growth delay *in vivo*. Click here for file
