# Supplementary material for: The alkylphospholipid, perifosine, radiosensitizes prostate cancer cells both in vitro and in vivo
Source: Radiat Oncol. 2011 Apr 15;6:39. doi: 10.1186/1748-717X-6-39 (PMC3096921; doi:10.1186/1748-717X-6-39)
Supplement: Additional file 1 — Figure S1: Radiosensitization of perifosine in prostate cancer PC-3 cells. Cells were irradiated in the absence (control) or in the presence of perifosine and the colony formation assay was conducted. Shown are the means and standard deviation of each individual treatment points. Figure S2: Perifosine and radiation induced apoptosis in PC-3 cells. Cells were treated with perifosine (5 μM), radiation, or combination as indicated. Cellular apoptosis was detected by FACs. Shown are the mean values of the quantitative data. Figure S3: Perifosine increases radiation induced tumor growth delay in vivo. [file 1748-717X-6-39-S1.PPT]

## Slide 1
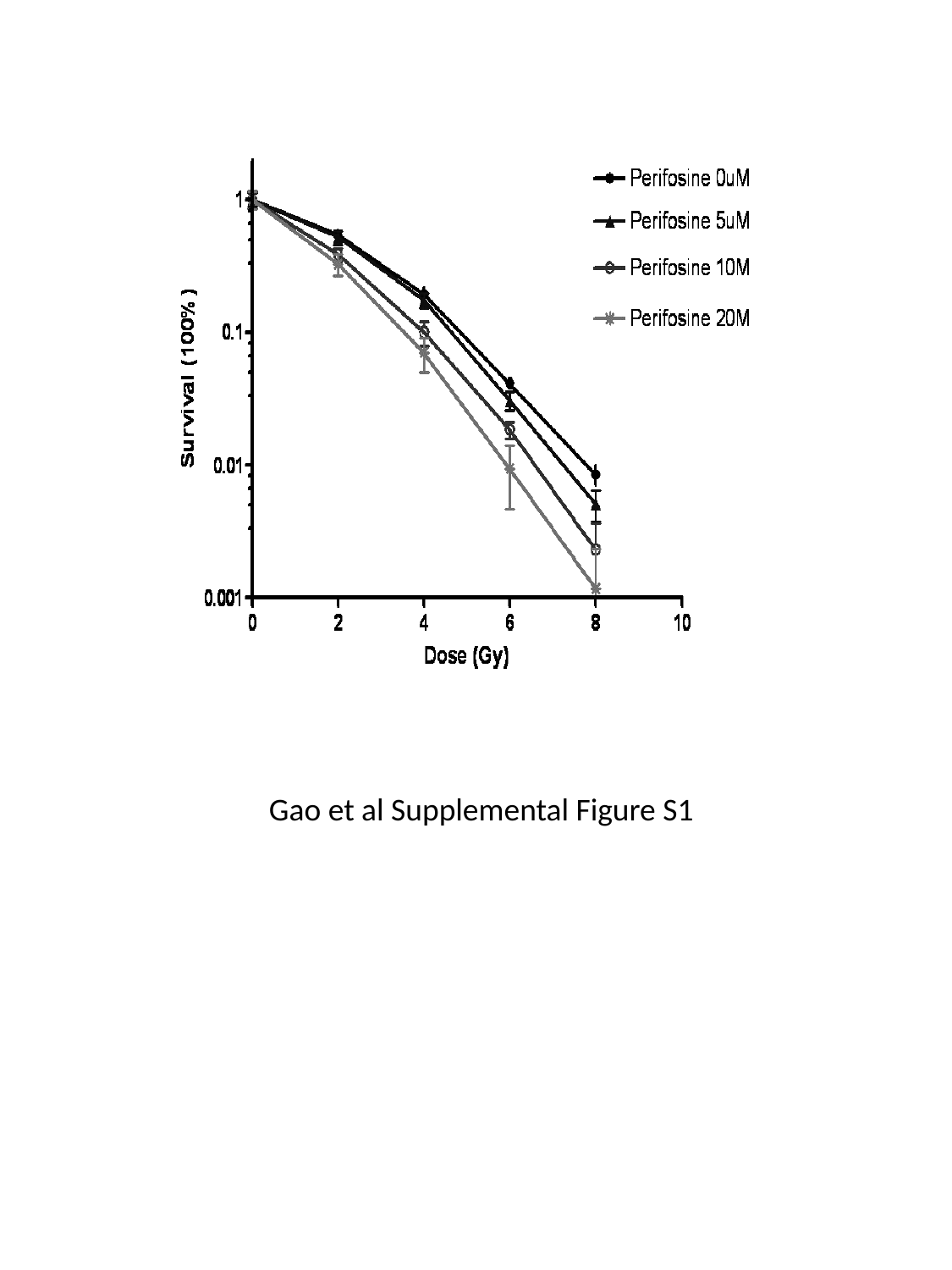

Gao et al Supplemental Figure S1

## Slide 2
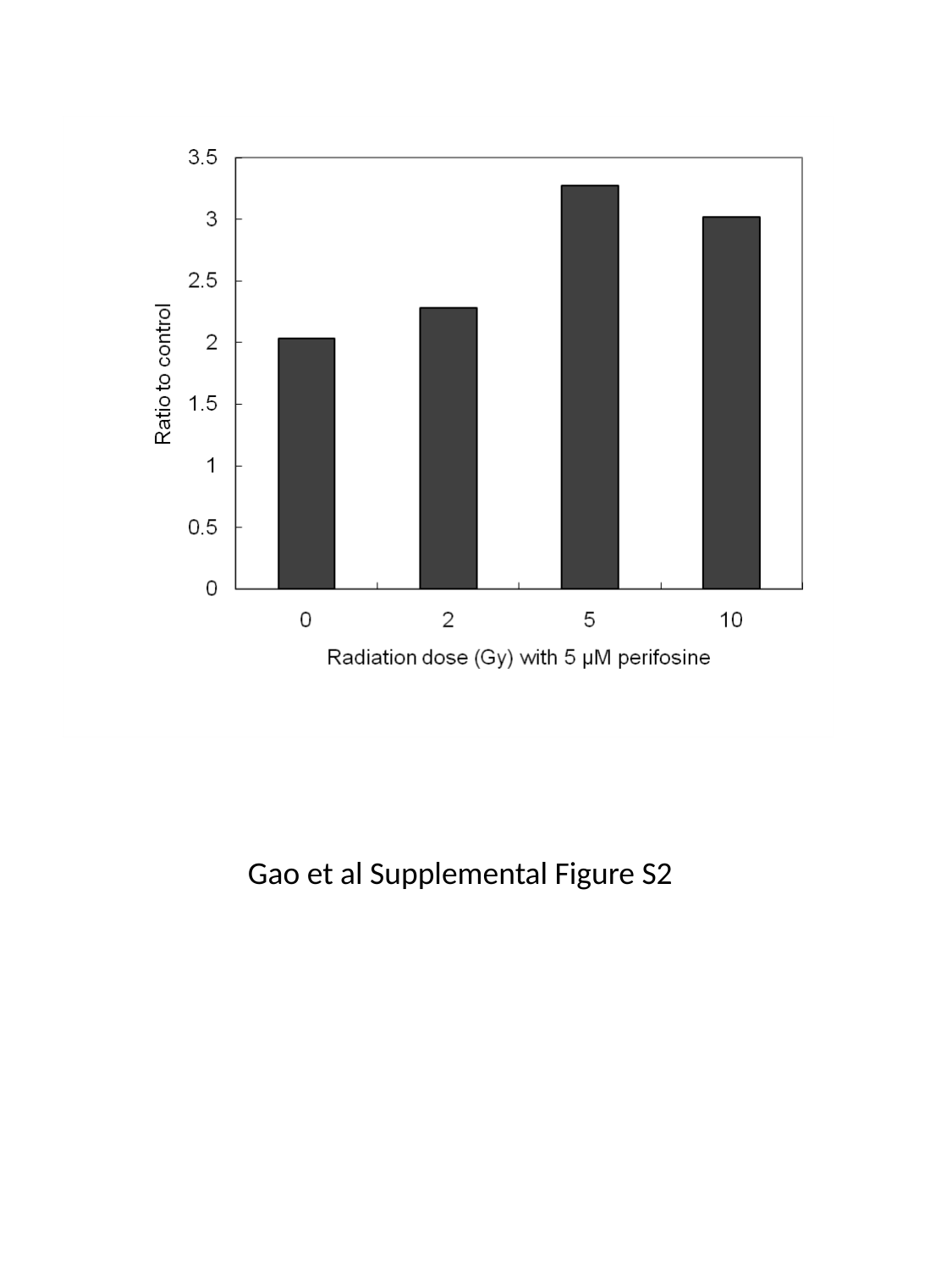

Gao et al Supplemental Figure S2

## Slide 3
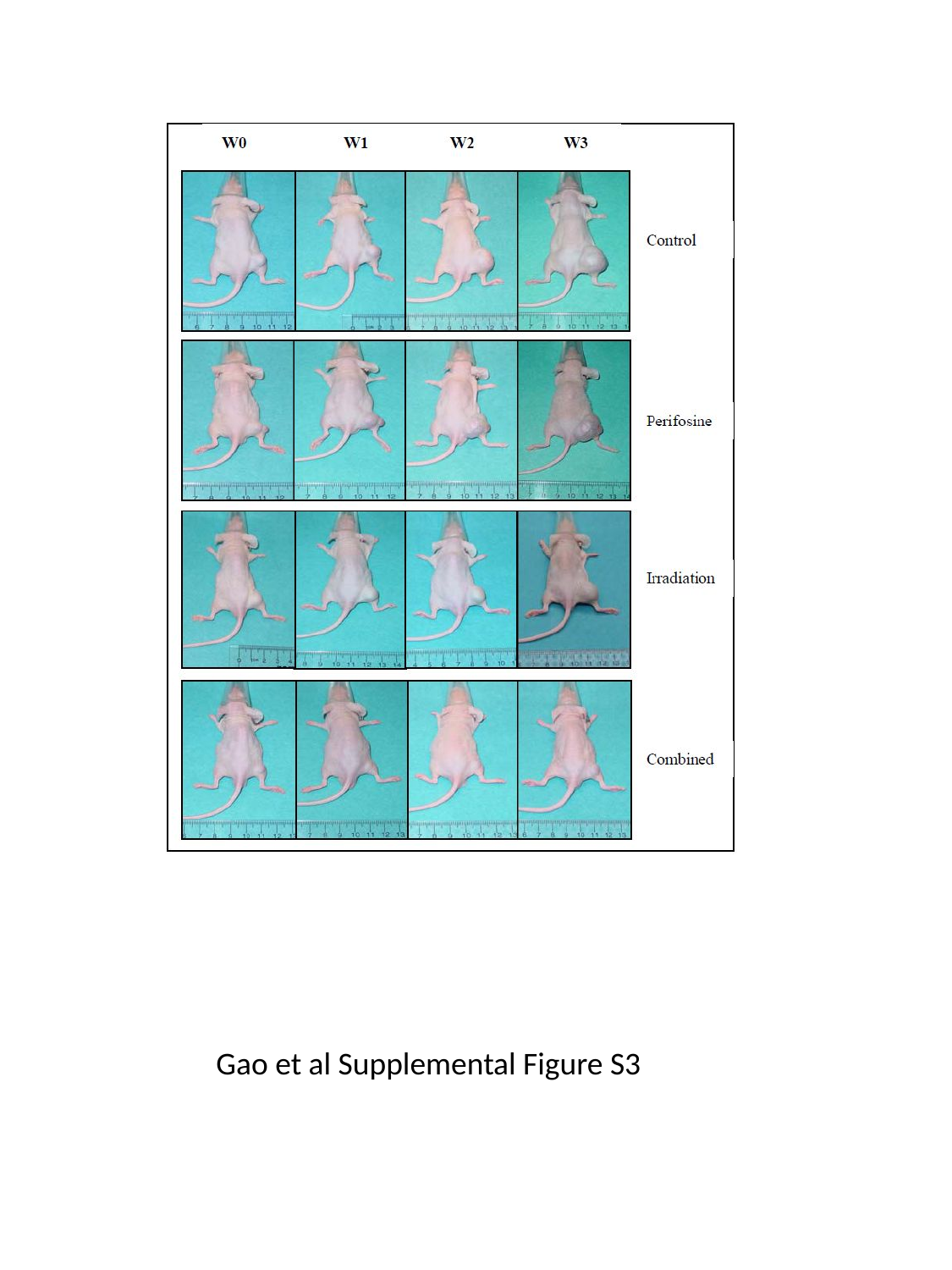

Gao et al Supplemental Figure S3
